# Pharmacological Properties of Chemically Characterized Extracts from Mastic Tree: In Vitro and In Silico Assays

**DOI:** 10.3390/life13061393

**Published:** 2023-06-14

**Authors:** Safae Ouahabi, El Hassania Loukili, Amine Elbouzidi, Mohamed Taibi, Mohammed Bouslamti, Hiba-Allah Nafidi, Ahmad Mohammad Salamatullah, Nezha Saidi, Reda Bellaouchi, Mohamed Addi, Mohamed Ramdani, Mohammed Bourhia, Belkheir Hammouti

**Affiliations:** 1 Laboratory of Applied and Environmental Chemistry (LCAE), Faculty of Sciences, Mohammed First University, B.P. 717, Oujda 60000, Morocco; 2 Laboratoire d’Amélioration des Productions Agricoles, Biotechnologie et Environnement (LAPABE), Faculté des Sciences, Université Mohammed Premier, Oujda 60000, Morocco; 3 Laboratories of Natural Substances, Pharmacology, Environment, Modeling, Health and Quality of Life (SNAMOPEQ), Faculty of Sciences, Sidi Mohamed Ben Abdellah University, Fez 30000, Morocco; 4 Department of Food Science, Faculty of Agricultural and Food Sciences, Laval University, Quebec City, QC G1V 0A6, Canada; 5 Department of Food Science & Nutrition, College of Food and Agricultural Sciences, King Saud University, P.O. Box 2460, Riyadh 11451, Saudi Arabia; 6 Laboratory of Bioresources, Biotechnology, Ethnopharmacology and Health, Faculty of Sciences, Mohammed First University, Boulevard Mohamed VI, B.P. 717, Oujda 60000, Morocco; 7 Department of Chemistry and Biochemistry, Faculty of Medicine and Pharmacy, Ibn Zohr University, Laayoune 70000, Morocco

**Keywords:** *P. lentiscus*, GC/MS, HPLC-DAD, antioxidant activity, antibacterial activity, antifungal activity, extract, molecular docking

## Abstract

The mastic tree, scientifically known as *Pistacia lentiscus*, which belongs to the Anacardiaceae family, was used in this study. The aim of this research was to analyze the chemical composition of this plant and assess its antioxidant and antibacterial properties using both laboratory experiments and computer simulations through molecular docking, a method that predicts the binding strength of a small molecule to a protein. The soxhlet method (SE) was employed to extract substances from the leaves of *P. lentiscus* found in the eastern region of Morocco. Hexane and methanol were the solvents used for the extraction process. The n-hexane extract was subjected to gas chromatography-mass spectrometry (GC/MS) to identify its fatty acid content. The methanolic extract underwent high-performance liquid chromatography with a diode-array detector (HPLC-DAD) to determine the presence of phenolic compounds. Antioxidant activity was assessed using the DPPH spectrophotometric test. The findings revealed that the main components in the n-hexane extract were linoleic acid (40.97 ± 0.33%), oleic acid (23.69 ± 0.12%), and palmitic acid (22.83 ± 0.10%). Catechin (37.05 ± 0.15%) was identified as the predominant compound in the methanolic extract through HPLC analysis. The methanolic extract exhibited significant DPPH radical scavenging, with an IC50 value of 0.26 ± 0.14 mg/mL. The antibacterial activity was tested against *Staphylococcus aureus*, *Listeria innocua*, and *Escherichia coli*, while the antifungal activity was evaluated against *Geotrichum candidum* and *Rhodotorula glutinis*. The *P. lentiscus* extract demonstrated notable antimicrobial effects. Additionally, apart from molecular docking, other important factors, such as drug similarity, drug metabolism and distribution within the body, potential adverse effects, and impact on bodily systems, were considered for the substances derived from *P. lentiscus*. Scientific algorithms, such as Prediction of Activity Spectra for Substances (PASS), Absorption, Distribution, Metabolism, Excretion (ADME), and Pro-Tox II, were utilized for this assessment. The results obtained from this research support the traditional medicinal usage of *P. lentiscus* and suggest its potential for drug development.

## 1. Introduction

The Anacardiaceae family encompasses the *Pistacia* genus, which comprises various plant species of notable significance in their food, medicinal, and ornamental properties [1]. The genus includes around 20 species, ranging from evergreen or deciduous trees, shrubs, and small trees, standing between 5 and 15 m in height [2,3]. *Pistacia* is widely distributed across regions such as Africa, Southern Europe, Asia, and North America. It mainly thrives in the Mediterranean region, where favorable humidity conditions exist for its growth [4]. *Pistacia* trees are dioecious, meaning they have male and female flowers growing on different trees [5]. Recently, the pharmaceutical industry has been interested in the ethnomedicinal and biological potentials of the *Pistacia* genus [1]. While the phytochemical composition of the genus has been widely studied, new research has been focusing on the therapeutic effects of its extracts for enhancing health [6,7,8,9,10,11,12,13,14].

The mastic tree, or *P. lentiscus*, is a shrub species belonging to the *Pistacia* genus. This evergreen bush has a characteristic scent and green leaves, growing in Mediterranean and Middle Eastern regions and reaching heights between 1 and 8 m [15]. Since ancient times, mastic tree extracts have been employed in folk medicine for their anti-inflammatory, antiseptic, and disease-treating properties, such as treating gastralgia and dyspepsia [16]. The aerial part of the mastic tree has been utilized as a stimulant and diuretic to treat hypertension [17]. Mastic tree products are widely used in the food industry due to their secondary metabolites, such as flavonoids, polyphenols, and phenolic acids. Recently, mastic tree extracts have been reported to exhibit antioxidant activity [17,18,19,20], and their use in cosmetic products has also been documented [21]. Additionally, this species has been shown to display antihepatotoxic, antibacterial, and antiproliferative properties in colon cancer cells [16,22,23].

**Figure 1:**
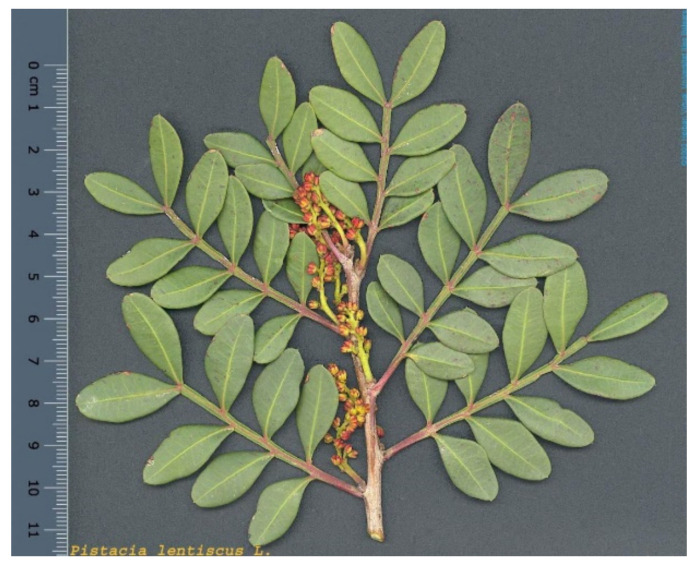
Leaves of *P. lentiscus*.

This investigation aimed to analyze the chemical constituents of *P. lentiscus* leaves (Figure 1) from the eastern region of Morocco. Furthermore, the extracts’ potential biological activities were assessed, explicitly focusing on the methanolic’s antimicrobial (antibacterial and antifungal) properties and antioxidant potential as measured by the DPPH free radical scavenging assay method. In addition, computational methods (molecular docking) were employed to investigate the principal compounds and their interactions, seeking to elucidate the underlying mechanisms.

## 2. Materials and Methods

### 2.1. Chemicals and Reagents

N-hexane and methanol were purchased from Merck (Darmstadt, Germany). 1,1-Diphenyl-2-picrylhydrazyl (DPPH•), Phenolic standards: Catechin, 4-hydroxybenzoic acid, *p*-Coumaric acid, naringin, quercetin, p-Coumaric acid, and luteolin were purchased from Merck and Carl Roth GmbH (Karlsruhe, Germany). All other chemicals used were of analytical grade.

### 2.2. Collection of Plant Material

Leaves of *P. lentiscus* were collected in March 2019 from Ahfir, located in the eastern region of Morocco. Subsequently, the leaves were meticulously cleansed and rinsed multiple times with distilled water, followed by air-drying in a well-ventilated location shielded from light and direct sunlight for 48 h. After that, lyophilization was carried out, and the leaves were ground into powder before extraction.

### 2.3. Soxhlet Extraction

A Soxhlet extraction apparatus consisting of a condenser, a Soxhlet chamber, and an extraction flask was used. In the extraction flask, 32 g of powder from dried mastic leaves were placed into an extraction thimble with 300 mL of the selected solvent (hexane and methanol). The period for the Soxhlet extractions experiments was chosen to be the time needed for the solvent to become colorless. After evaporation under vacuum, the extracts obtained were named HEPL and MEPL, respectively.

### 2.4. Determination of Extraction Yield

The extracts were stored in a refrigerated environment for preservation purposes. The extraction yield, expressed as a percentage, was calculated by dividing the weight of the obtained extract (*M extract*) by the weight of the dried initial sample (*M dry matter*) used for the extraction process. This calculation was carried out following the formula specified in the corresponding equation:

$$Extraction\;yield\;\left(\%\right)=\left[\frac{M\;extract\;\left(g\right)}{M\;dry\;matter\;\left(g\right)}\right]\times 100$$

### 2.5. Fatty Acid GC-MS Analysis of P. lentiscus Extracts

A modified version of the Fatty Acid GC-MS Analysis of *P. lentiscus* Extracts to analyze the fatty acid content of the hexane extract of *P. lentiscus* protocol as described by Loukili et al. [24] was employed. The BPX25 capillary column coupled to a QP2010 MS from Kyoto, Japan, was utilized in identifying and separating the fatty acids. Pure helium gas was the carrier gas at a constant flow rate of 3 mL/min. The temperature of the injection, ion source, and interface was maintained at 250 °C, while the temperature of the column oven was gradually increased from 50 °C to 250 °C at a rate of 10 °C/min. The ionization of sample components was performed in the EI mode (70 eV) with a mass range scanned of 40–300 *m*/*z*. The extract, diluted in n-hexane, was injected in a split mode with a volume of 1 μL, and the sample was analyzed in triplicate. The compounds were identified by comparing their retention times with authentic standards and their mass spectral fragmentation patterns with those stored in databases or on the National Institute of Standards and Technology (NIST). LabSolutions software, version 2.5, was utilized for data collection and processing.

### 2.6. Identification of Phenolic Compounds by HPLC-DAD

The HPLC/DAD Waters Corporation USA was employed to analyze the methanol extract. The separation module of the liquid chromatography (Waters; e2695) was coupled with a diode array detector (Waters 2998; PDA), and Empower software was used for data processing. A mobile phase gradient mode was applied on the AC18 column (5 μm, 4.6 mm × 250 mm), and the resulting chromatogram was captured using wavelengths in the 254–300 nm range. The mobile phase was composed of solvent A (ultrapure water/acetic acid, 2% *v*/*v*) and solvent B (acetonitrile) with varying compositions over time: 0–5 min: 95% A and 5% B; 25–30 min: 65% A and 35% B; 35–40 min: 30% A and 70% B; 40–45 min: 95% A and 5% B. The sample (20 μL) was injected, and the flow rate was set at 0.9 mL/min. Standard polyphenolic compounds like catechin, p-Coumaric acid, 4-hydroxybenzoic acid, Coumaric acid, quercetin, luteolin, and naringenin were utilized to identify the peaks by matching their retention times and UV spectra.

### 2.7. Antioxidant Activity

#### 2.7.1. Determination of Antioxidants by DPPH Radical Scavenging Activity

The objective of the present investigation was to evaluate the antioxidant potential of the methanolic extract derived from the foliage of *P. lentiscus*. The antioxidant activity was determined using the DPPH radical scavenging assay, wherein ascorbic acid was used as a reference compound. In brief, 0.8 mL of the sample or standard solution at different concentrations (0.5, 0.4, 0.3, and 0.1 mg/mL) was mixed with 2 mL of DPPH• solution (4 mg of DPPH• dissolved in 200 mL of ethanol) and then mixed manually. Following this, the samples were kept in the dark at room temperature for 30 min, and their absorbance was measured at λ of 517 nm with a reference blank. This procedure was performed in triplicate to ensure reproducibility, and the reduction in DPPH• absorbance was recorded. Subsequently, the percentage of DPPH• radical scavenging inhibition by the sample was calculated using the following equation:

$$Inhibition\;\left(\%\right)=\left[\frac{\left(Ab-As\right)}{Ab}\right]\times 100$$

The determination of the extract concentration leading to 50% *inhibition* (IC50) involved the plotting of the inhibition percentage against the extract concentration on a graph. The absorbance of the blank (*Ab*) and that of the positive control or sample (*As*) were utilized in this process.

#### 2.7.2. β-Carotene Bleaching Assay

The antioxidant activity of *P. lentiscus* extracts was evaluated using the β-Carotene Bleaching assay. The emulsion was prepared by dissolving 2 mg of β-carotene, 20 mg of linoleic acid, and 200 mg of Tween 80 in 10 mL of chloroform. The solution was evaporated at 40 °C under a vacuum, and 100 mL of distilled water was added while stirring vigorously. The emulsion was mixed with either the extract or a reference antioxidant (BHA) at a concentration of 1 mg/mL in separate test tubes to evaluate the antioxidant activity. The absorbance at 470 nm was measured using a 96-well microplate reader at two different times: immediately after adding the emulsion (t0) and after 2 h. The volume of the emulsion used was 0.2 mL.

### 2.8. Antimicrobial Activity

#### 2.8.1. Bacterial Strains

In this investigation, three bacterial strains, namely *Escherichia coli* (ATCC 10536), *Staphylococcus aureus* (ATCC 6538), and *Listeria innocua* (ATCC 49.189) were utilized as model organisms to assess the effectiveness of *P. lentiscus* extract as a bacterial growth inhibitor. The bacterial strains were obtained from the Microbiology and Microbial Biotechnology Laboratory of the Faculty of Science in Oujda, Morocco. The Mueller Hinton agar medium was used to cultivate bacterial cultures, which were kept at 37 °C for 24 h. The concentration of bacterial cells was determined to be 106 cells/mL using a UV-visible spectrophotometer at a wavelength of 620 nm before assessing the inhibitory effects of the *P. lentiscus* extract.

#### 2.8.2. Agar-Diffusion Method

To qualitatively evaluate the antimicrobial efficacy of the substance against microbial strains, the agar diffusion method, a widely accepted technique for assessing susceptibility to various microbial strains, was used. The National Clinical Laboratory Standards Committee’s guidelines were adhered to while carrying out the method. This method efficiently evaluates a substance’s capacity to inhibit bacterial strains’ proliferation [25]. The process included the addition of prepared bacterial inoculum to Petri dishes containing agar growth medium (MHA), where wells were made using a Pasteur pipette filled with 50 μL of the extract being tested. These plates were then incubated at 37 °C for 48 h, and the size of the inhibitory zone was measured to determine antimicrobial activity. Each test was conducted in triplicate to ensure the results’ accuracy.

#### 2.8.3. Determination of MIC and MBC

In assessing the effectiveness of antimicrobial agents, the determination of minimum inhibitory concentration (MIC) is critical. The present study utilized the resazurin microtiter assay to evaluate the MIC of *P. lentiscus* extract [26]. In this assay, a colorimetric indicator called resazurin is employed. It is reduced by metabolically active cells, which results in a color shift from blue to pink. Each well of a 96-well microplate contained different concentrations of the antimicrobial agent, and a standardized inoculum of the test bacteria was added to each well during the assay. The microplates were then incubated at 37 °C for 24 h and added resazurin to each well. A further 4–6 h of incubation occurred until a color change was observed. The MIC was determined as the lowest concentration of the antimicrobial agent that resulted in no color change, indicating the absence of viable bacteria. Controls were included in each microplate to confirm the accuracy of the results. The MBC was determined by inoculating a volume of 3 μL was taken from the negative wells as a sample, which was plated onto Mueller Hinton Agar medium plates and incubated at 37 °C for 24 h. The extract’s lowest concentration that did not result in bacterial growth determined the MBC. The experiment was repeated in triplicate to ensure reproducibility.

### 2.9. Antifungal Activity

#### 2.9.1. Selection of Fungal Strains for Antifungal Activity Testing Using *P. Lentiscus* Extract

In this study, two distinct fungal strains, namely *Rhodotorula glutinis* (ON 209167) and *Geotrichum candidum*, were selected to evaluate the antifungal potential of *P. lentiscus* extract. The strains were obtained from the Microbiology and Biotechnology Laboratory of the Faculty of Sciences in Oujda, Morocco.

#### 2.9.2. Agar Diffusion Method

Culture conditions for the two fungal strains, *G. candidum* and *R. glutinis*, were optimized before testing the antifungal activity of *P. lentiscus* extract. *G. candidum* was cultured on potato dextrose agar medium for seven days at 25 °C, and the resulting spore concentration was adjusted to 2 × 10^6^ spores/mL using the Thoma cell hemocytometer. *R. glutinis*, on the other hand, was cultured on Yeast Extract Peptone Dextrose for 48 h at 25 °C, and the cell concentration was measured and adjusted to 10^6^ cells/mL. To test the antifungal activity of the *P. lentiscus* extract, the agar diffusion method, as used for bacteria, was employed with a slight modification in the culture medium (Yeast Extract Glucose). This method is widely used for testing susceptibility to different fungal strains and was performed according to the guidelines established by the National Clinical Laboratory Standards Committee [25].

#### 2.9.3. Determination of MIC and MBC

The determination of MIC is crucial in evaluating the efficacy of antifungal agents. In this investigation, the resazurin microtiter test was utilized to determine the MIC of *P. lentiscus* extract [27]. The test was carried out in 96-well microplates, each containing a concentration range of 16% to 0.25%. A standardized inoculum of the fungal strain was added to each well, followed by incubation at 25 °C for 48 h. After incubation, resazurin was added, and the microplates were further incubated for 2 h until a color change from blue to pink was observed. The MIC was defined as the lowest concentration of the antifungal agent that did not result in a color change, indicating the absence of viable fungi. Positive and negative controls were included in each microplate to confirm the accuracy of the results. The MBC was determined by inoculating a 3 μL sample from the negative wells onto YEG and PDA medium plates, which were then incubated at 25 °C for 48 to 72 h. The MFC corresponded to the extract’s lowest concentration, which did not result in any observable growth.

### 2.10. ADME and Toxicity Prediction

To determine the pharmacokinetic characteristics of the compounds being studied, this research assessed their profile in terms of Absorption, distribution, metabolism, and excretion (ADME). Computational tools, like SwissADME and pkCSM web servers, were utilized to forecast these characteristics. The examination assessed various physical and chemical traits of the compounds, their similarity to drugs, and their pharmacokinetic properties. These properties included their capacity to penetrate cell membranes, interact with transporters and enzymes responsible for drug absorption and elimination, and their metabolic stability [28,29,30]. Moreover, the toxicity levels of the molecules were estimated using the Protox II online tool [30]. This tool employs a statistical algorithm that compares the chemical structure of a substance to a vast database of toxic compounds to predict the probability of the substance causing harmful effects or toxicity to both humans and other living organisms. By utilizing the Protox II tool, significant details concerning the toxicity class, LD50 values, and toxicological endpoints like immunotoxicity, mutagenicity, cytotoxicity, hepatotoxicity, and carcinogenicity were obtained. As a result, these advanced computational techniques and instruments provided valuable comprehension of the potential therapeutic uses and risks of toxicity associated with the identified compounds.

### 2.11. PASS Prediction

To evaluate the pharmacological activity and potential toxicity risks of the primary chemical constituents present in Artemisia species’ essential oils, we employed a set of sophisticated computational methods and tools [31]. Our methodology comprised the utilization of the PASS (Prediction of Activity Spectra for Substances) technique, which involves a statistical calculation that compares the chemical composition of a particular compound with an extensive database of bioactive compounds. This process allows for the forecast of the biological outcomes of these compounds, including enzyme inhibition, receptor binding, and alteration of metabolic pathways [32,33]. With the use of this approach, it becomes possible to predict the probability of a substance exhibiting specific activities. The first step involved converting the substances to SMILES format through ChemDraw. The converted compounds were then analyzed using the PASS web application, which provides insight into the possible activity (Pa) and inactivity (Pi) of drug-like compounds before the evaluation.

### 2.12. Molecular Docking Analysis

**Figure 2:**
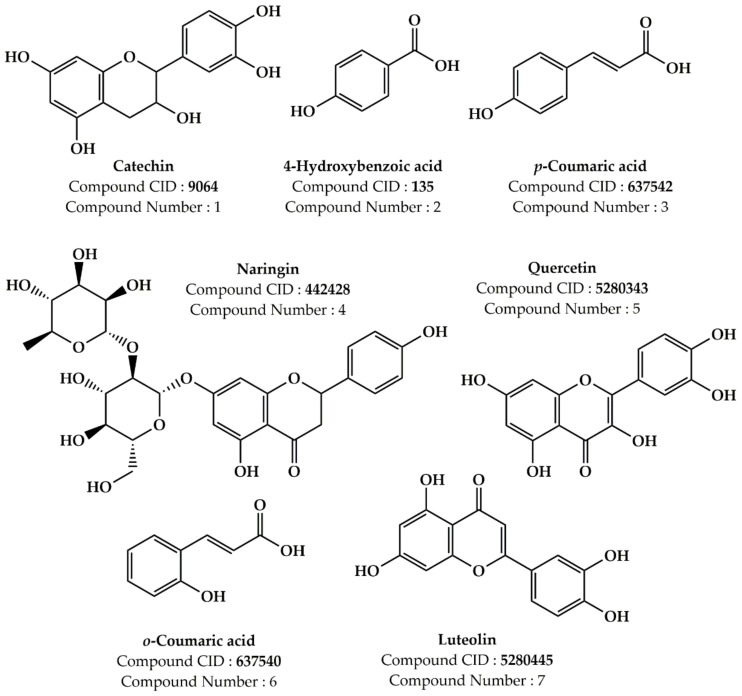
The molecular structure of the components found in *P. lentiscus* samples.

To explore the possible therapeutic characteristics of *P. lentiscus* extract, we employed molecular docking techniques to anticipate the antioxidant, antibacterial, and antifungal features of the seven phytocompounds present in the extract (Figure 2, Table 1). Our research approach was based on established methods previously described in the literature [34,35,36,37,38,39]. We acquired the three-dimensional (3D) configurations of the molecules from PubChem in March 2023 and converted them into a “pdb” file via the PyMol program. To examine how these plant compounds interact with specific proteins, we acquired the protein structures from the Protein Data Bank website using their distinctive PDB IDs. The protein structures were subjected to standard procedures, including the removal of inhibitors, water molecules, and ions and the addition of polar H-bonds and Kollmann charges to improve the accuracy of the protein structures. For automated docking experiments, we used AutoDock Vina v1.5.6 software and AutoGrid to generate grid maps that displayed the interaction energy between the ligands and target proteins during the docking process [40]. We expanded the search space grid box to enhance the accuracy of the docking procedure (Table 2). The ligand complex binding energies (∆G) were denoted in Kcal/mol, and we created two-dimensional (2D) diagrams with the Discovery Studio 4.1 program. The interactions were then subject to further analysis.

## 3. Results

### 3.1. Extraction Yield

The extraction yields of *P. lentiscus* extracts are presented in Table 3. To extract dried leaves of *P. lentiscus*, the efficiency of various solvents was examined, and the findings demonstrate that methanol (17.5 ± 0.05%) had the highest yield among the solvents tested. In contrast, hexane exhibited a low extraction yield (6.37 ± 0.13%).

### 3.2. Fatty Acid Analysis

**Figure 3:**
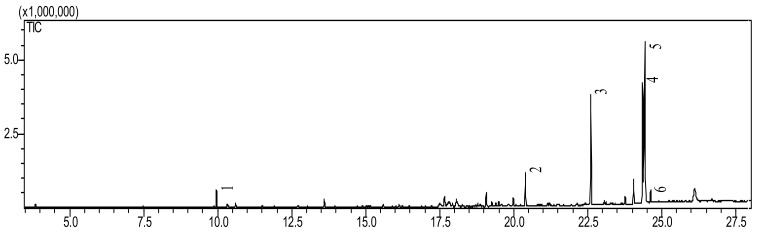
Chromatogram GC/MS of hexanic extract from *P. lentiscus.* (1) D-Limonene, (2) Myristic Acid (C14:0), (3) Palmitic Acid (C16:0), (4) Oleic Acid (C18:1), (5) Linoleic Acid (C18:2), and (6) 10-methyl-Heptadecanoic acid.

**Figure 4:**
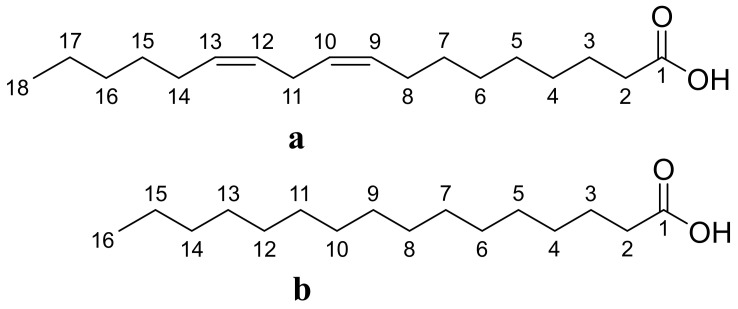
Main compounds of hexanic extract: (**a**) linoleic acid (C18:2), (**b**) palmitic acid (C16:0).

The hexanoic extract from dried mastic leaves of *P. lentiscus* was analyzed by Gas chromatography coupled with mass spectrometry (GC–MS) to identify the profile of volatile compounds (Figure 3). The results are given in Table 4. The research findings showed that *P. lentiscus* contains a variety of fatty acids, with a notably high concentration of C18 USFA and C16 SFA (Figure 4), such as C18:2, C18:1, and C16:0. Specifically, the analysis identified six compounds, including C18:2 linoleic acid (making up 40.9% of the fatty acids), C18:1 oleic acid (23.6%), C16:0 palmitic acid (22.8%), C14:0 myristic acid (6.3%), D-Limonene (3.56%), and 10-methyl-Heptadecanoic acid (1.29%) like are shown in Table 4.

### 3.3. HPLC-DAD Analysis

**Figure 5:**
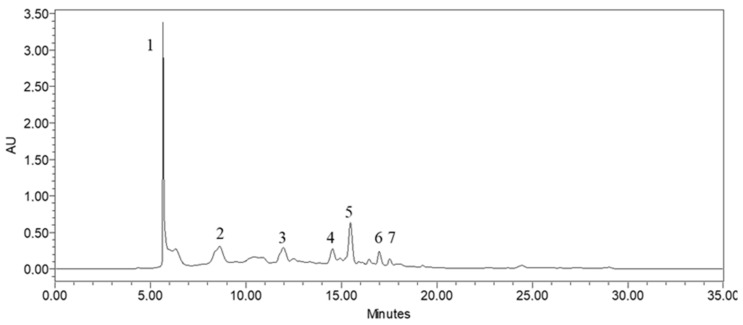
HPLC profile of the methanolic extract of *P. lentiscus*.

**Figure 6:**
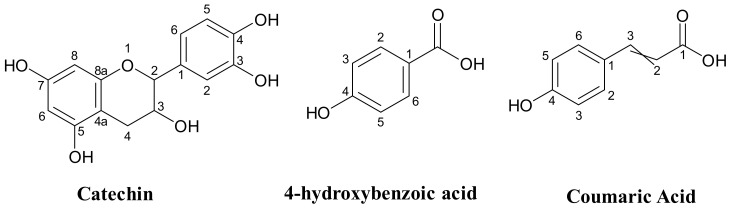
Most abundant compounds detected of *P. lentiscus* leaves methanolic extract.

The phytochemical study of the methanolic extract obtained from *P. lentiscus* was assessed using HPLC/DAD (high-performance liquid chromatography/diode-array detector). To determine the components within the extract, their retention times and UV spectra were compared to those of the corresponding standards. Figure 5 presents the HPLC chromatogram, which shows the identified polyphenolics, while Figure 6 exhibits the chemical structure of the main components found in the extract.

The methanolic extract was found to contain several phenolic compounds, detectable at 254 nm as shown in Table 5. These compounds’ highest concentration was catechin, accounting for approximately 37% of the total compounds detected. Other notable compounds present were 4-hydroxybenzoic acid and Coumaric acid, which accounted for around 17% of the total, followed by naringin at 8.9%, p-Coumaric acid at 7.1%, quercetin at 6.7%, and luteolin at 4.8%.

### 3.4. Antioxidant Activity

To assess the antioxidant potential of both the methanolic extract of *P. lentiscus* and the standard (ascorbic acid), the DPPH radical scavenging assay and β-carotene bleaching assay were conducted. The first test measures the reduction of the DPPH radical, which is reflected in a change in color from purple (DPPH•) to yellow (DPPH-H) upon adding an antioxidant. The test used a spectrophotometer to measure the absorbance at 515 nm, while the second test, the β-carotene bleaching assay, is used to evaluate a substance’s antioxidant capacity. In this assay, β-carotene is mixed with linoleic acid and exposed to oxidative conditions. Antioxidants within a test substance can prevent or slow the degradation of β-carotene and linoleic acid, which results in a decreased rate of color fading, indicating a higher antioxidant capacity. The outcomes of the assay are presented in Table 6.

Based on current research, the methanolic extract derived from *P. lentiscus* leaves exhibited potent free radical scavenging ability, as indicated by its IC_50_ value of 0.26 ± 0.13 mg/mL. This value is comparable to that of the reference standard, ascorbic acid, which had an IC_50_ value of 0.15 ± 0.11 mg/mL. These findings align with those reported by Zitouni et al. [43], who reported an IC_50_ value of 0.166 mg/mL for *P. lentiscus* from Algeria, and by Hemma et al. [44], who reported an IC_50_ value of 0.121 ± 0.001 mg/mL. Additionally, the extract demonstrated high β-carotene bleaching activity with an IC_50_ value of 0.19 ± 0.26 mg/mL, similar to the reference antioxidant BHA. In contrast, the hexanic extract exhibited IC_50_ values higher than the reference antioxidants, ascorbic acid, and BHA.

### 3.5. Antibacterial Activity

The objective of this study was to investigate the antibacterial effects of *P. lentiscus*, a medicinal and aromatic plant, on Gram-positive bacteria (*Staphylococcus aureus* and *Listeria innocua*) and a Gram-negative bacterium (*Escherichia coli*). The diameters of the inhibition halos surrounding the discs were measured using a graduated ruler (Table 7) to determine the aortograms, and the minimum inhibitory concentration (MIC) and minimum bactericidal concentration (MBC) of the extract were assessed using the microdilution technique. The study revealed that *P. lentiscus* extracts had different antibacterial properties against the bacterial strains tested, with growth inhibition zone diameters ranging from 7 to 20 mm. *Staphylococcus aureus* exhibited the largest inhibition zone diameter (IZ) of 20 mm, while *E. coli* exhibited the smallest IZ of 7 mm. Specifically, a concentration of 2% was shown to be effective against *Staphylococcus aureus*. In addition, the extract inhibited the growth of *Listeria innocua* at concentrations of 4% and 8%.

### 3.6. Antifungal Activity

The extract exhibited moderate antifungal activity against the tested fungal species, with growth inhibition zones of 22 mm and 12 mm observed for *R. glutinis* and *G. candidum*, respectively (Table 8). The extract demonstrated a minimum inhibitory concentration value of 8% and a minimum fungicidal concentration of approximately 16% against *G. candidum*.

### 3.7. Physiochemical and ADME Prediction Analysis

The results of the molecular analysis and the drug-likeness analysis of the chosen molecules are summarized in Table 9. The properties listed include molecular weight (MW), topological polar surface area (TPSA), hydrogen bond donors (H-Bonds), hydrogen bond acceptors, and the number of rotatable bonds. Additionally, the table shows whether the molecules violated Lipinski’s rule of five [45], Ghose filter [46], and Veber filter [47]. Overall, the chosen small molecules have a range of properties, and some violate certain drug-likeness filters. Catechin, quercetin, and *o*-Coumaric acid have good drug-likeness properties as they do not violate any of the filters.

In contrast, naringin and luteolin violate multiple filters and are less likely to be considered potential drugs. 4-Hydroxybenzoic acid violates the Ghose filter but is accepted by the Lipinski and Veber filters. The molecular weight of the molecules ranges from 138.12 g/mol for 4-hydroxybenzoic acid to 580.53 g/mol for naringin, with the majority of molecules having a molecular weight of less than 350 g/mol. The TPSA values range from 57.53 Å^2^ for 4-hydroxybenzoic acid and p-Coumaric acid to 225.06 Å^2^ for naringin, with the majority of molecules having TPSA values less than 140 Å^2^.

**Figure 7:**
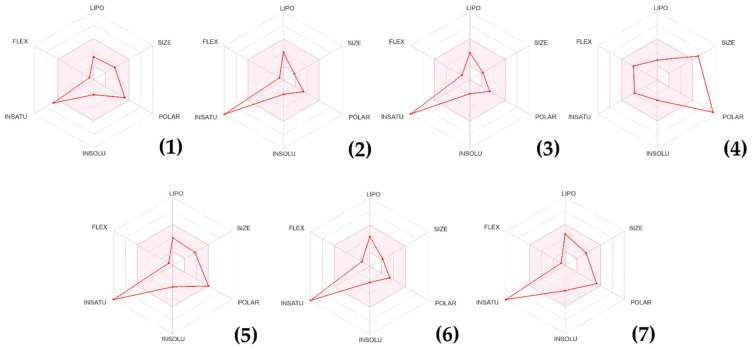
Bioavailability radars of *P. lentiscus* extract’s molecules. (**1**) Catechin, (**2**) 4-Hydroxybenzoic acid, (**3**) p-Coumaric acid, (**4**) naringin, (**5**) quercetin, (**6**) o-Coumaric acid, (**7**) luteolin.

Table 9 presents the pharmacokinetic properties of identified compounds in *P. lentiscus* extract, which could provide insights into their potential use as therapeutic agents. The table includes absorption parameters, such as bioavailability score, water solubility, Caco-2 permeability, and intestinal absorption; distribution parameters, such as solubility class, skin permeation (log Kp), the Volume of Distribution at steady-state (VDss), and Blood–Brain Barrier (BBB) permeability, metabolism parameters such as CYP2D6 and CYP3A4 substrate and inhibitor, and excretion parameters such as total clearance and renal Organic Cation Transporter 2 (OCT2) substrate. In terms of absorption parameters, the bioavailability score ranges from 0.17 to 0.85, indicating that some compounds may be well absorbed while others may have lower bioavailability (Figure 7). The Caco-2 permeability values suggest that most of the compounds have moderate to high permeability, with one compound having low permeability. The intestinal absorption percentages range from 25.79% to 93.49%, with most compounds having high absorption rates. Regarding distribution parameters, all compounds are soluble, and the log Kp values suggest that they are likely to distribute into tissues. The VDss values suggest that some compounds may have limited tissue distribution while others may distribute widely. Additionally, BBB permeability indicates that most compounds are likely to cross the BBB. The metabolism parameters suggest that none of the compounds are substrates or inhibitors of CYP2D6 or CYP3A4, which are important enzymes involved in drug metabolism. Finally, the excretion parameters suggest that most compounds are eliminated primarily via hepatic clearance, with total clearance values varying from 0.183 to 0.746 mL/min/kg. None of the compounds are substrates of renal OCT2. Overall, these results provide insight into the pharmacokinetic properties of the identified compounds in *P. lentiscus* extract, which could be useful for predicting their potential efficacy and safety in various applications.

**Figure 8:**
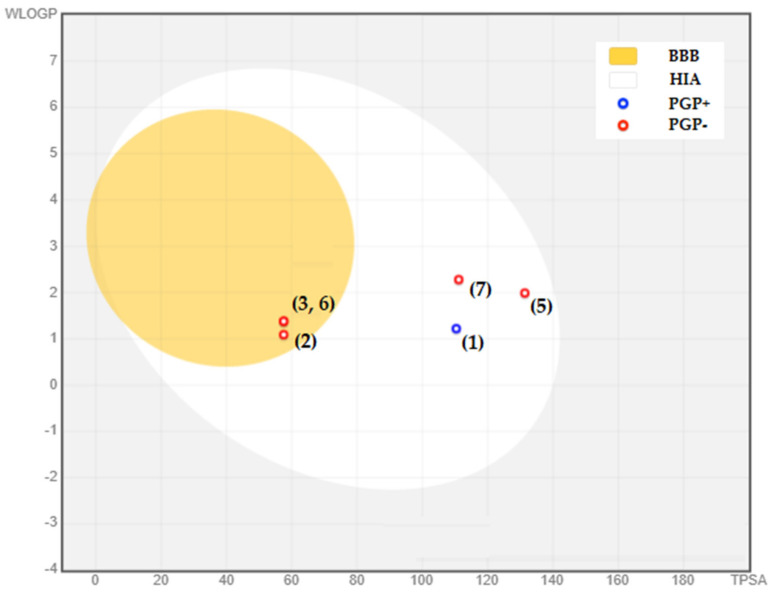
Boiled-egg model of the identified molecules in *P. lentiscus* extract. (1) Catechin, (2) 4-Hydroxybenzoic acid, (3) p-Coumaric acid, (5) quercetin, (6) o-Coumaric acid, (7) luteolin. Remark: naringin (4) was found to be out of range.

The BOILED-Egg model is based on the molecule’s lipophilicity (WLOGP) and polarity (TPSA), which are two essential characteristics that play a critical role in determining a molecule’s behavior in the body [48]. The BOILED-Egg model visually represents these characteristics, with the white area indicating molecules likely to be absorbed by the intestines and the yellow area indicating high potential for the molecule to penetrate the blood–brain barrier. In a recent study on *P. lentiscus* phytocompounds, the BOILED-Egg model was used to evaluate their potential to be absorbed by the intestines and penetrate the blood-brain barrier (Figure 8). Three compounds, 4-Hydroxybenzoic acid, p-Coumaric acid, and O-Coumaric acid, have a high ability to be absorbed by the intestines and penetrate the BBB while also being non-substrates of the P-glycoprotein (P-GP), which is a protein that can prevent certain molecules from entering the brain. On the other hand, compounds 1, 5, and 7 (catechin, quercetin, and luteolin, respectively) were found to have a poor ability to penetrate the brain endothelial cells. P-GP was found to be able to transport catechin as a substrate, meaning it may have difficulty entering the brain due to P-GP’s actions. However, the other two compounds were identified as non-substrates of P-GP, indicating they may still have some potential to penetrate the blood-brain barrier. One compound, naringin (4), was found to be out of range due to its high TPSA value. This indicates that naringin may not be an effective candidate for penetrating the blood–brain barrier due to its high polarity.

### 3.8. Toxicity Prediction Using Pro-Tox II Webserver

The Pro Tox-II server was used to examine the toxicity profile of chemicals derived from *P. lentiscus*, and Table 10 provides a summary of the results. Based on the findings, none of the chemicals derived from *P. lentiscus* were found to have the potential to induce hepatotoxicity or cytotoxicity., indicating that they were all rather safe to use. Four of the studied substances, notably *p*-Coumaric acid, quercetin, *o*-Coumaric acid, and luteolin, were shown to be possibly carcinogenic in terms of their ability to induce cancer. Nonetheless, it was found that the likelihood of their occurrence was less than 0.68, indicating a negligibly low propensity to cause cancer. Contrarily, the immunotoxicity of naringin (4) was determined with a prediction probability of 0.99, demonstrating the substance’s potential to cause immunotoxicity. Regarding the mutagenicity of the tested compounds, only two compounds, quercetin (5) and luteolin (7), were identified as being potentially mutagenic with a probability of 0.51. These findings highlight the importance of assessing natural compounds’ safety and toxicity profiles before utilizing them for various applications, particularly in medicine and the food industry, to ensure their safe and effective use.

### 3.9. PASS Prediction and Molecular Docking Analysis

#### 3.9.1. PASS Prediction

Prediction of Activity Spectra for Substances (PASS) involves a series of algorithms that utilize structural data about the molecule to predict its activity against a wide range of biological targets, including enzymes, receptors, and ion channels [49,50]. By utilizing a vast database of known active and inactive chemicals, statistical models are developed that can accurately predict the activity of newly developed compounds [49]. In the early stages of drug discovery, PASS analysis is a widely used tool to prioritize compounds for further testing and to guide the design of new compounds with optimal activity profiles. As depicted in Table 11, PASS predicted activity (P act) and predicted inactivity (P ina) values for seven different compounds, namely catechin, 4-Hydroxybenzoic acid, p-coumaric acid, naringin, quercetin, o-Coumaric acid, and luteolin, were assessed across three categories of biological activity, namely antioxidant, antibacterial, and antifungal. For the antioxidant activity, it appears that compounds 1, 4, 5, and 7 (catechin, naringin, quercetin, and luteolin) have relatively high P act values (above 0.75), suggesting they may have antioxidant activity. Compounds 2, 3, and 6 (4-Hydroxybenzoic acid, *p*-Coumaric acid, and *o*-Coumaric acid) have lower P act values, indicating a lower likelihood of antioxidant activity. For the antibacterial and antifungal activities, the predictions revealed a potent antifungal activity for compound (4) Naringin with a P act = 0.816.

#### 3.9.2. Molecular Docking Results

Molecular docking is a commonly used methodology employed mainly in drug design that is based on molecular structure. Its development in the 1980s has been instrumental in drug discovery, as it enables the prediction of small-molecule ligand placement in target binding sites and can help stabilize ligand–receptor complexes through the investigation of molecular events, such as ligand-protein binding [51,52].

The docking scores, represented as binding energies (in kcal/mol), of the seven identified compounds from *P. lentiscus* against six proteins with antibacterial, antifungal, and antioxidant properties are shown in Table 12. The docking scores of the native ligands are also provided as a reference point. A heat map is used to visualize the data, with red indicating a stronger binding affinity than the native ligand, yellow indicating a similar affinity, and green indicating a weaker affinity.

Molecular docking can help stabilize ligand–receptor complexes. In this study, we have used molecular docking to investigate how *P. lentiscus* extract components work. The resulting binding affinity values indicate whether the molecule has a higher or lower affinity for the target compared to a known inhibitor. The interactions of these components have been investigated with specific enzymatic proteins, including lipoxygenase-3 (PDB ID: 1N8Q) [53,54] and cytochrome P450 (PDB ID: 1OG5) [53,55], which are known target receptors for antioxidant chemicals. For proteins known to have bactericidal/bacteriostatic activity, DNA Gyrase Topoisomerase II and Enoyl-Acyl Carrier Protein Reductase were selected as therapeutic targets (PDB IDs: 1KZN and 3GNS, respectively) [36,38,53]. Furthermore, the investigation focused on two proteins associated with antifungal activity, namely Cytochrome P450 14 alpha-sterol Deme-thylase (PDB ID: 1EA1) and N-Myristoyl transferase (PDB ID: 1IYL) [53].

The bacterial enzyme DNA gyrase topoisomerase II (PDB: 1KZN) is ubiquitously present in all bacterial species and regulates the topological state of bacterial DNA. In this study, we identified the enzyme DNA gyrase topoisomerase II, which consists of two subunits, GyrA and GyrB, as the target protein for binding. The compounds extracted from *P. lentiscus* exhibited weak binding affinity with this protein, except for naringin, which demonstrated a binding score ranging from −5.6 to −9.3 kcal/mol, with a score of −9.3 kcal/mol. When protein 1KZN was docked with its natural ligand clorobiocin, the outcome demonstrated strong inhibitory potential, as evidenced by the docking score of −9.6 kcal/mol. The ligand established two conventional H-bonds with the amino acid residues of the active site, THR165, and ASP73, which was mentioned in the reference [53]. Our docking analysis revealed the presence of four potent inhibitors of FabI activity (PDB ID: 3GNS), namely quercetin, catechin, luteolin, and naringin, with binding scores of −7, −7.1, −7.2, and −8.1 kcal/mol, respectively, compared to triclosan, a well-known FabI inhibitor, with a score of −6.2 kcal/mol.

The formation and upkeep of bacterial cell membranes require fatty acid biosynthesis, which involves a sequence of enzyme-catalyzed reactions that transform fundamental components into long, unsaturated fatty acids, constituting a significant part of these structures. The final stage of this process is the reduction of the double bond in an intermediate molecule referred to as an enoyl-acyl carrier protein (ACP) derivative [56]. The enzyme FabI, also known as enoyl-ACP reductase, is responsible for facilitating the reduction process in the final stage of fatty acid biosynthesis. It plays an essential role in the elongation phase of this process, which is necessary for the cycle’s completion [57]. FabI has been identified in crystal structures of various bacteria, including *Escherichia coli* and *Staphylococcus aureus* [58].

The N-terminal glycine of various proteins in eukaryotic organisms is bound by N-Myristoyl Transferase (NMT), which utilizes myristoyl-CoA as a substrate and attaches myristate fatty acid [59]. NMT is essential for various crucial biological processes, including cell death, signal transduction, and the proliferation of fungal pathogens [60]. The study revealed that catechin, naringin, quercetin, and luteolin are potent inhibitors of the second lipoxygenase member, CYP2C9, with binding affinity values lower than −6.6 kcal/mol, the value of the ligand warfarin.

The biosynthesis of sterols in fungi depends heavily on the enzyme CYP51s, also referred to as Cytochrome P450 14α-Sterol Demethylase. This enzyme is responsible for producing intermediary compounds required for the formation of ergosterol, making it a critical component of the process [61]. Due to its indispensable role in the formation of sterols, CYP51s have become a prime aim for antifungal drugs. In this study, molecular docking analysis was carried out using the crystal structure of CYP51s (with a PDB ID of 1EA1) as a basis. The results of the analysis indicate that the molecules quercetin, catechin, luteolin, and naringin possess strong inhibitory potential with docking scores of −7.1, −6.6, −7.3, and −9.1 kcal/mol, respectively.

The current study also shows that the same four molecules, quercetin, catechin, luteolin, and naringin, exhibit potent inhibitory potential against NMT, with binding affinities higher than that of Fluconazole, a known antifungal drug with a docking score of −5.8 kcal/mol.

Lipoxygenases are a group of enzymes that use a redox mechanism to catalyze the oxidation of polyunsaturated fatty acids, producing hydroperoxide, which is an oxygen-centered radical that can be involved in the development of severe diseases.

In this study, two proteins were selected, namely lipoxygenase (1N8Q) and cytochrome P450 (1OG5). The study identified catechin, O-Coumaric acid, luteolin, and quercetin as potent inhibitors of lipoxygenase (PDB ID: 1N8Q), with binding affinity scores of −6.3, −6.3, −6.3, and −8.3 kcal/mol, respectively, compared to the inhibitor Protocatechuic Acid, which had a docking score of −6.0 kcal/mol.

## 4. Discussion

In this research, extracts were prepared from the leaves of *P. lentiscus*. Several studies [62,63] have confirmed that the yield depends on the polarity of the solvent used, which is consistent with our study. In line with Ariana Bampouli et al.’s study [64], our results demonstrate that hexane is not an appropriate solvent for extracting metabolites from mastic tree leaves due to its low extraction efficiency. On the other hand, ethanol or methanol as solvents led to significantly higher yields. Generally, non-polar solvents like hexane extract non-polar compounds like carotenes, terpenes, and lipids, while polar compounds like phenolic compounds are typically extracted using water, ethanol, or methanol [65].

The chemical composition of H.E was determined by gas chromatography-mass spectrometry (GC-MS). The primary compound detected in the *P. lentiscus* extract was linoleic acid, which is a precursor for arachidonic acid biosynthesis and serves as a substrate for synthesizing eicosanoids. According to previous research, linoleic acid has potential cholesterol-lowering effects and can be beneficial for the skin, making it a widely used ingredient in the cosmetic industry [66,67]. The *P. lentiscus* extract is rich in unsaturated fatty acids (UFA), specifically C18:1 (MUFA) and C18:2 (PUFA), accounting for around 64.67% of its content. These levels suggest that the extract is resistant to oxidation and could serve as a valuable source of MUFA and PUFA in the diet. MUFA is essential for its nutritional implications and impact on the extract’s oxidative stability. Previous studies have shown that a diet high in MUFA may be a healthier alternative to a low-fat diet, as it can lower blood cholesterol levels, modulate immune function, decrease the susceptibility of LDL to oxidation, and enhance the fluidity of HDL [68]. Furthermore, including PUFA in the diet is crucial for the structure and function of membrane proteins, such as receptors, enzymes, and active transport molecules [69].

The major phenolic compounds of the M.E were determined through HPLC analysis. It revolves around the presence of catechins as major compounds. Catechins are flavan-3-ols, a natural polyphenolic compound belonging to the flavonoid family, found in numerous plants. These compounds possess various biochemical properties and exhibit antioxidant activity, which can prevent diseases such as cancer, cardiovascular, and neurodegenerative diseases by reducing oxidative stress [70].

The antioxidant activity was analyzed using two methods (the DPPH radical scavenging method and the β-carotene bleaching assay). The potent scavenging activity of the methanolic extract from *P. lentiscus* leaves is believed to be attributed to its high concentration of phenolic compounds, particularly catechin. The present discovery corresponds with the investigation conducted by Luo and colleagues [71], where they proposed that dissimilarities in the number of phenolic compounds among diverse plant species can elucidate the discrepancies in their antioxidant activity.

The antibacterial and antifungal activities of *P. lentiscus* methanolic extract were analyzed. The antibacterial potency of extracts from medicinal and aromatic plants depends on the diversity of the molecules present [72]. These results suggest that moderate concentrations of the extract may efficiently inhibit the growth and spread of the tested bacterial strains. The antibacterial activity of *P. lentiscus* is attributed to its chemical composition, particularly the presence of phenolic compounds [73]. Catechin, the primary compound found in this plant, is thought to be responsible for the antibacterial effects [74], while naringin and other compounds can also contribute to the activity against Gram-positive bacteria [75]. The activity of the extract against Gram-negative bacteria is comparatively lower than that against Gram-positive bacteria due to the differences in the composition of their cell walls. In Gram-positive microorganisms, the resistance is attributed to the presence of a hydrophilic barrier that hinders diffusion through the outer cell membrane. However, in Gram-negative bacteria, the inhibition of activity is primarily based on the direct interaction between the extract’s hydrophobic components and the phospholipids present in the cell membrane. This interaction can lead to structural damage and complete rupture of the cell membranes [50].

The growth-inhibitory properties of *P. lentiscus* extract against these fungi are attributed to its chemical composition. The antifungal potential of plant-derived substances has been widely investigated through in vitro studies [76]. This moderate activity is a result of several factors acting together to achieve the antifungal effect. The extract’s chemical composition and its richness in bioactive constituents are considered to be important determinants of antifungal activity. Notably, the presence of flavonoids is responsible for the extract’s antifungal properties by disrupting the fungal cell membrane, degrading the mitochondrial function, inhibiting electron transport, and altering mitochondrial ATPase activity [77]. On the other hand, the extract was found to be inactive against molds, such as *R. glutinis*, which could be attributed to the inherent resistance of molds to the components present in the sample.

A drug needs to exhibit high selectivity and minimal side effects [78]. However, despite being effective, many potential treatments do not make it through the later stages of testing due to their high rate of attrition caused by unavoidable side effects [78]. These side effects can arise due to the complexity of the biological system and can be difficult to predict. To reduce the risk of drug candidate failure, in silico studies can be conducted to assess drug-likeness and ADME properties of potential molecules [45,46]. After the initial screening process, the identified molecules undergo a thorough analysis to determine their drug-like properties and ADME profiles. This comprehensive analysis can help to identify potential issues and minimize the risk of a drug candidate failing in the later stages of testing.

Lipinski’s rule of five is a widely used rule to predict drug-likeness, and violations of this rule suggest that the molecule may have poor Absorption or permeation in the body. The Ghose and Veber filters are other commonly used filters to predict drug-likeness. Among the molecules listed, catechin, 4-Hydroxybenzoic acid, *p*-Coumaric acid, quercetin, and o-Coumaric acid have good drug-like properties, with no violations of any filters. These molecules may have the potential for drug development. Naringin and luteolin have multiple violations of drug-likeness filters, indicating that they may have poor pharmacokinetic properties and are less likely to be developed as drugs. However, it is important to note that the results of these filters are not absolute and do not entirely rule out the possibility of these molecules being developed as drugs.

The BOILED-Egg model is an effective and convenient tool to evaluate the potential for a substance to be absorbed by the intestines and to penetrate the blood–brain barrier [48]. It was able to provide valuable insights into the potential of *P. lentiscus* phytocompounds to be absorbed by the intestines and penetrate the BBB, which can be useful for further drug development and therapeutic applications.

Prediction of Activity Spectra for Substances (PASS) is a computational approach extensively utilized in drug research to predict the biological activity of chemical compounds [49]. The results of this analysis indicate that some compounds have relatively modest to high P act values for one category but not for the other, which may suggest that any noticed biological activity may be attributed to a potential synergy.

Molecular docking, a widely used methodology in drug design, was employed in this study to investigate the interactions between the components of *P. lentiscus* extract and specific enzymatic proteins. The binding affinity values obtained from the docking analysis provided insights into the potential inhibitory activity of the compounds. For DNA Gyrase Topoisomerase II, most compounds from *P. lentiscus* showed weak binding affinity, except for naringin, which demonstrated strong binding affinity. The natural ligand clorobiocin exhibited potent inhibitory potential, establishing two conventional H-bonds with the amino acid residues of the active site. In the case of Enoyl-Acyl Carrier Protein Reductase (FabI), four compounds from *P. lentiscus*, namely quercetin, catechin, luteolin, and naringin, showed potent inhibitory activity, suggesting their potential as FabI inhibitors. These compounds displayed higher binding scores compared to the well-known FabI inhibitor Triclosan. N-Myristoyl Transferase (NMT) inhibition was observed with catechin, naringin, quercetin, and luteolin, indicating their potential as inhibitors for this enzyme. These compounds exhibited higher binding affinities than the antifungal drug Fluconazole.

The results also revealed the strong inhibitory potential of quercetin, catechin, luteolin, and naringin against Cytochrome P450 14α-Sterol Demethylase (CYP51s), an enzyme crucial for sterol biosynthesis in fungi. These compounds displayed significant binding scores, indicating their potential as antifungal agents targeting CYP51s. Lastly, lipoxygenase-3 (1N8Q) inhibition was observed with catechin, O-coumaric acid, luteolin, and quercetin, suggesting their potential as lipoxygenase inhibitors. These compounds showed higher binding affinities compared to the inhibitor Protocatechuic Acid. Overall, the results of this study highlight the potential of the identified compounds from *P. lentiscus* extract as inhibitors for enzymes involved in antibacterial, antifungal, and antioxidant activities. Further investigations and experimental validations are warranted to confirm their efficacy and suitability as therapeutic agents.

## 5. Conclusions

Our research aims to improve the extracts derived from *P. lentiscus* in the Ahfir region of eastern Morocco. Through the analysis of the hexanic extract using GC/MS, we have identified linoleic acid as the major fatty acid compound, comprising approximately 40% of the extract. The unsaturated fatty acid to saturated fatty acid ratio is 2.12%, indicating a high content of unsaturated fatty acids, which are valuable in the food industry. The chemical composition of the methanolic extract was determined using HPLC-DAD, revealing that catechin is the predominant compound, accounting for around 37% of the extract. The methanolic extract demonstrated antioxidant activity close to that of ascorbic acid when assessed using the DPPH radical scavenging method. However, as these extracts are crude and contain various compounds, purified compounds may exhibit comparable activity to ascorbic acid. Our findings indicate that *P. lentiscus* extracts possess significant antimicrobial properties, as demonstrated by both in vitro and in silico assays, suggesting their potential as a phytomedicine. The in silico assays supported the experimental data and provided insights into the molecular interactions between the tested drugs and specific enzymatic proteins, indicating substantial binding affinities. In conclusion, our research has shown that the studied products exhibit excellent antioxidative and antibacterial properties, positioning them as potential candidates for various biotechnological applications.

## Figures and Tables

**Table 1:** Characteristics of the chosen small molecules for the docking study and their drug-likeness properties.

Molecules	MW * (g/mol)	TPSA (Å^2^)	H-Bonds		Rotatable Bonds	Lipinski’s Rule of Five (Violations)	Ghose Filter (Violations)	Veber Filter (Violations)
			Acceptors	Donors				
Catechin	290.27	110.38	6	5	1	Yes	Yes	Yes
4-Hydroxybenzoic acid	138.12	57.53	3	1	1	Yes	No (3 violations: MR < 40, MW < 160, number of atoms < 20)	Yes
*p*-Coumaric acid	164.16	57.53	3	1	2	Yes	Yes	Yes
Naringin	580.53	225.06	14	8	6	No (3 violations: MW > 500, N or O > 10, NH or OH > 5.)	No (4 violations: MW > 480, WLOGP < −0.4, MR > 130, number of atoms > 70)	No (1 violation: TPSA > 140)
Quercetin	302.24	131.36	7	5	1	Yes	Yes	Yes
*o*-coumaric acid	164.16	57.53	3	1	2	Yes	Yes	Yes
Luteolin	286.24	111.13	6	3	1	No (3 violations: MW > 500, N or O > 10, NH or OH > 5.)	No (4 violations: MW > 480, WLOGP < −0.4, MR > 130, number of atoms > 70)	No (1 violation: TPSA > 140)

* MW: molecular weight; MR: molar refractivity.

**Table 2:** Molecular modeling targets and grid box characteristics.

Proteins/PDB IDs	Native Ligand	Grid Box Size (x, y, z)/Center (x, y, z)	Reference
DNA Gyrase Topoisomerase II (*E. coli*)/1KZN	Clorobiocin	(40, 40, 40)/(19.528, 19.500, 43.031)	[38]
Enoyl-Acyl Carrier Reductase Protein/3GNS	Triclosan	(40, 40, 40)/(−14.280, 0.562, −21.462)	[35,36]
Cytochrome P450 14 Alpha-Sterol Demethylase/1EA1	Fluconazole	(40, 40, 40)/(17.702, −3.978, 67.221)	[39,41]
N-Myristoyl Transferase/1IYL	Fluconazole	(40, 40, 40)/(−11.256, 49.991, 1.040)	[39,41]
Lipoxygenase/1N8Q	Protocatechuic Acid	(40, 40, 40)/(22.455, 1.293, 20.362)	[42]
CYP2C9/1OG5	Warfarin	(12.387, 11.653, 11.654)/(−19.823, 86.686, 38.275)	[42]

**Table 3:** Extraction yield.

Extracts	Yield (%)	
	This Work	Literature
HEPL ^1^	6.37 ± 0.13	2.00 ± 0.10
MEPL ^2^	17.5 ± 0.05	13.10 ± 0.91

^1^: Hexanic extract of *P. lentiscus* leaves (HEPL), ^2^: Methanolic extract of *P. lentiscus* leaves (MEPL).

**Table 4:** Chemical composition of hexane extract from *P. lentiscus*. ^a^: Unsaturated Fatty Acids (UFA); ^b^: Saturated Fatty Acids (SFA); ^c^: ratio UFA/SFA.

Compounds	TR (min)	HEPL (%)
D-Limonene	9.870	3.57 ± 0.12
Myristic Acid (C14:0)	20.392	6.39 ± 0.15
Palmitic Acid (C16:0)	22.608	22.84 ± 0.20
Oleic Acid (C18:1)	24.433	23.70 ± 0.13
Linoleic Acid (C18:2)	24.600	40.97 ± 0.11
10-methyl-Heptadecanoic acid	24.625	1.29 ± 0.01
UFA ^a^	64.670	
SFA ^b^	30.516	
UFA/SFA ^c^	2.120	

**Table 5:** Phenolic compound of methanolic extract of *P. lentiscus* leaves.

Compounds	T_R_ (min)	MEPL (%)
Catechin	6.077	37.045 ± 0.15
4-hydroxybenzoic acid	8.527	17.763 ± 0.21
p-Coumaric acid	10.418	7.107 ± 0.10
Naringin	11.965	8.914 ± 0.09
Quercetin	14.539	6.739 ± 0.06
Coumaric Acid	15.473	17.589 ± 0.11
Luteolin	16.978	4.839 ± 0.02

**Table 6:** Antioxidant activity of methanolic extract of *P. lentiscus*.

Antioxidant Activity	DPPH	β-Carotene
	Inhibitory Concentration 50 (μg/mL)	
HEPL	0.58 ± 0.73	0.64 ± 0.5
MEPL	0.26 ± 0.13	0.19 ± 0.26
Ascorbic Acid	0.15 ± 0.11	
BHA	0.09 ± 0.15	

**Table 7:** Exploring the MIC and the MBC concentrations of *P. lentiscus* extract.

Bacterial Strains	Inhibition Zone (mm)		*P. lentiscus* Extract		
	*P. lentiscus* Extract	Gentamicin (1 mg/mL)	MIC (%)	MBC (%)	MBC/MIC
*S. aureus*	20.00	19.50	2	4	2
*L. innocua*	15.00	21.50	4	8	2
*E. coli*	07.00	20.50	>16	>16	-

**Table 8:** Exploring the MIC and the MFC concentrations of *P. lentiscus* extract against fungal strains.

Fungi Strains	Inhibition Zone (mm)		*P. lentiscus* Extract		
	*P. lentiscus* Extract	Cycloheximide (1 mg/mL)	MIC (%)	MFC (%)	MFC/MIC
*G. candidum*	12.00	23.00	8	16	2
*R. glutinis *	22.00	21.00	>16	>16	-

**Table 9:** Pharmacokinetic properties of the identified compounds in *P. lentiscus* extract.

Prediction	1	2	3	4	5	6	7
ADME Prediction Absorption Parameters							
Bioavailability score	0.55	0.85	0.55	0.17	0.55	0.85	0.55
Water Solubility (log mol/L)	−3.117	−1.877	−2.378	−2.919	−3.221	−1.56	−3.294
Caco-2 Permeability	−0.283	1.151	1.21	−0.658	−0.057	1.158	0.286
Intestinal Absorption (%)	68.82	83.96	93.49	25.79	75.34	91.11	82.17
**Distribution**							
Class of solubility	Soluble	Soluble	Soluble	Soluble	Soluble	Soluble	Soluble
Log K*_p_* (cm/s)	−7.82	−6.02	−6.26	−10.15	−7.05	−5.86	−6.25
VDss (log L/kg)	1.027	−1.557	−1.151	0.619	−0.03	−0.406	−0.173
BBB Permeability	No	Yes	Yes	No	No	Yes	No
**Metabolism**							
CYP2D6, and CYP3A4 Substrate	No	No	No	No	No	No	No
CYP2D6, and CYP3A4 Inhibitors	No	No	No	No	No	No	Yes
**Excretion**							
Total Clearance log (mL/min/kg)	0.183	0.593	0.662	0.318	0.484	0.746	0.568
Renal OCT2 Substrate	No	No	No	No	No	No	No

**Table 10:** The toxicological characteristics of compounds derived from the methanolic extract of *P. lentiscus* leaves were evaluated using Pro-Tox II. (1) Catechin, (2) 4-Hydroxybenzoic acid, (3) p-Coumaric acid, (4) naringin, (5) quercetin, (6) o-Coumaric acid, (7) luteolin.

	Predicted LD_50_ (mg/kg)	Class	Hepatotoxicity		Carcinogenicity		Immunotoxicity		Mutagenicity		Cytotoxicity	
			Predi. *	Prob.	Predi.	Prob.	Predi.	Prob.	Predi.	Prob.	Predi.	Prob.
1	10,000	VI	In.	0.72	In.	0.51	In.	0.96	In.	0.55	In.	0.84
2	2200	V	In.	0.52	In.	0.51	In.	0.99	In.	0.99	In.	0.86
3	2850	V	In.	0.52	Ac.	0.50	In.	0.91	In.	0.93	In.	0.81
4	2300	V	In.	0.81	In.	0.80	Ac.	0.99	In.	0.73	In.	0.66
5	159	III	In.	0.69	Ac.	0.68	In.	0.87	Ac.	0.51	In.	0.99
6	2850	V	In.	0.52	Ac.	0.50	In.	0.91	In.	0.93	In.	0.81
7	3919	V	In.	0.69	Ac.	0.68	In.	0.97	Ac.	0.51	In.	0.99

* Predi.: Prediction, Prob: Probability, In.: Inactive, Ac.: Active.

**Table 11:** PASS prediction of the identified phytocompounds. (1) Catechin, (2) 4-Hydroxybenzoic acid, (3) *p*-Coumaric acid, (4) naringin, (5) quercetin, (6) *o*-Coumaric acid, (7) luteolin. P act: probable activity; P ina: probable inactivity.

Prediction	1	2	3	4	5	6	7
	PASS Prediction (P act/P ina)						
Antioxidant	0.810/0.003	0.320/0.020	0.553/0.005	0.851/0.003	0.872/0.003	0.553/0.005	0.775/0.004
Antibacterial	0.320/0.053	0.384/0.034	0.343/0.045	0.669/0.005	0.387/0.033	0.343/0.045	0.388/0.033
Antifungal	0.552/0.023	0.384/0.053	0.451/0.039	0.816/0.004	0.490/0.032	0.451/0.039	0.520/0.027

**Table 12:** Heatmap displaying the docking scores (affinity values in kcal/mol) of components from *P. lentiscus*.

N°	Compounds	Antibacterial Proteins		Antifungal Proteins		Antioxidant Proteins	
		1KZN	3GNS	1EA1	1IYL	1N8Q	1OG5
		Docking Scores (Kcal/mol) *					
-	Native Ligand	−9.6	−6.2	−5.8	−5.8	−6	−6.6
1	Catechin	−8.3	−7.1	−6.6	−7	−6.3	−8.3
2	4-Hydroxybenzoic acid	−5.6	−5.5	−4.5	−4.7	−5.4	−5.3
3	*p*-coumaric acid	−5.8	−5.3	−4.6	−5.4	−5.7	−6.2
4	Naringin	−9.3	−8.1	−9.1	−7.7	−5.6	−7.8
5	Quercetin	−8.3	−7	−7.1	−7.1	−8.3	−8.8
6	*o*-coumaric acid	−6.1	−5.7	−4.7	−5.6	−6.3	−5.9
7	Luteolin	−8.9	−7.2	−7.3	−7.2	−6.3	−8.8

* Each column is assigned a color scale ranging from red, which represents the docking score of the native ligand (∆G), to green, which represents a higher binding affinity than the native ligand (∆G + 4 kcal/mol). The midpoint is represented by the color yellow.

## Data Availability

Not applicable.
